# Supplementary material for: Topological state-space estimation of functional human brain networks
Source: PLoS Comput Biol. 2024 May 13;20(5):e1011869. doi: 10.1371/journal.pcbi.1011869 (PMC11115255; doi:10.1371/journal.pcbi.1011869)
Supplement: Attachment — Submitted filename: response.PLOScomputational.2023.12.11.pdf [file pcbi.1011869.s001.pdf]

Response Letter for the 1st Revision

Manuscript ID: PCOMPBIOL-D-23-01171
Persistent Homological State-Space Estimation
of Functional Human Brain Networks at Rest

We thank the editors and reviewers for constructive comments and corrections that improved the quality of revision substantially. We addressed every comment and question very carefully in the resubmission. The edited parts as well as new materials are colored in blue in the main manuscript. The major changes are all listed in this response letter. Minor changes such as additional new references and corrections are not listed.

Reviewer 1

R1-1 *There are too few references in the last 3 years, please update more recent literatures in the introduction and discussion sections.*

Thank you for suggestion. We did additional literature review between years 2000-2023, and added many new references. Following paragraphs in Introduction and discussion have been added or revised accordingly. Also not listed here, additional new references are added through the paper.

INTRODUCTION:

Unlike traditional graph theory approaches that analyze networks at a single fixed scale, persistent homology examines networks across multiple scales. It identifies topological features that remain persistent and are robust against different scales and noise perturbations [Petri et al., 2014, Sizemore et al., 2018, 2019, Vaccarino et al., 2022]. Recent studies have further illustrated the versatility of persistent homology in analyzing complex networks, including brain networks. Sizemore et al. [2019], Xing et al. [2022] highlighted the application of persistent homology in evaluating temporal changes in topological network features. Aktas et al. [2019] used persistent homology to detect and track the evolution of networks' clique. Billings et al. [2021] discussed the use of simplicial complexes encoded by persistent homology for brain networks. Sizemore et al. [2018] applied persistent homology to investigate the spatial distributions of cliques and cycles in brain networks. Khalid et al. [2014], Caputi et al. [2021] showed how persistent homology could be used in the analysis of functional brain connectivity using EEG data. Chung et al. [2023] utilized persistent homology

to analyze brain networks for studying abnormal white matter in maltreated children. These studies collectively emphasize the potential of persistent homology in providing a robust framework for multiscale network analysis. This approach's ability to capture topological features across different scales and under varying conditions makes it particularly suitable for studying the complex brain networks.

While Euclidean loss remains the dominant cost function in deep learning, topological losses based on persistent homology are emerging as superior in tasks requiring topological understanding [Chen et al., 2019, Hu et al., 2019, Gupta et al., 2022, Lin et al., 2023]. These topological losses incorporate penalties based on the topological features of the data, distinguishing them from the Euclidean loss, which primarily focuses on differences at the node or edge level. By encoding the intrinsic topological structure of the network, topological losses facilitate the creation of more informative feature maps, potentially enhancing overall model performance [Hofer et al., 2019]. Gupta et al. [2022] demonstrated that image segmentation based on topological loss outperforms other deep learning architectures for similar tasks. Lin et al. [2023] introduced a new architecture that excels in segmenting curvilinear structures by learning topological similarities over existing methods.

DISCUSSION:

In this study, we proposed the topological clustering method for the estimation and quantification of dynamic state changes in time-varying brain networks. A coherent statistical theory, grounded in persistent homology, was developed, and we demonstrated the application of this method to resting-state fMRI data. Resting-state brain networks tend to persist in a single state for extended periods before transitioning to another state [Allen et al., 2014, Shakil et al., 2016, Calhoun and Adali, 2016, Rosenberg et al., 2020]. The average brain network in each state, as depicted in Figure 10, appears to diverge from the patterns reported in previous studies [Cai et al., 2018, Haimovici et al., 2017, Huang et al., 2020]. Further research is required for independent validation.

In contrast to previous studies that reported relatively low heritability in functional brain networks [Glahn et al., 2010, Xu et al., 2017, Korgaonkar et al., 2014, Wan et al., 2022], our findings indicate significant higher heritability across various regions of the brain network. This discovery not only challenges the prevailing understanding but also opens new avenues for exploring genetic influences on brain network dynamics. Our observations align with the early findings by Lykken et al. [1982], which documented higher heritability in EEG spectra. This parallel suggests that heritability in brain networks may be more nuanced than previously understood. The distinction in our approach lies in its methodology: rather than directly using the connectivity strength, we decomposed networks into discrete topological states and computed heritability for each state. This granular analysis provides a more accurate estimation of heritability across different functional states of the brain. The resting state measures employed

in studies such as those by Glahn et al. [2010], Xu et al. [2017], Korgaonkar et al. [2014] directly rely on *static* connectivity matrices. These matrices, while informative, often do not capture the dynamic and configural nature of brain networks. Such methods may overlook hidden configural patterns that hold significant heritable information. Our topological method represents a significant advancement in this regard. By focusing on the topological aspects of dynamic brain networks, our method is adept at identifying and extracting hidden patterns of high heritability that might be missed by traditional approaches. This capability could be crucial for understanding the genetic basis of various neuropsychiatric and neurodevelopmental disorders, where altered brain network configurations play a critical role.

Intraclass correlation (ICC) has long been recognized as a vital reliability and reproducibility metric, especially for gauging similarity in paired data when the order of pairing is not preserved [Chen et al., 2018, Sarker et al., 2023, Solís-Lemus et al., 2023]. In brain imaging, it serves as a popular baseline for test-retest (TRT) reliability assessments, often in conjunction with the Dice coefficient [Liao et al., 2013, Cole et al., 2014, Cousineau et al., 2017, Pfaehler et al., 2021, Zhang et al., 2018]. The widespread use of ICC in these contexts underscores its perceived utility in evaluating consistency across imaging sessions or different imaging modalities. The conventional computation of ICC is typically through an ANOVA statistical model, which can be fairly limited and inflexible. Recent years have seen a shift towards mixed-effects models, which offer greater flexibility and accuracy in estimating ICC, especially in datasets with nested or hierarchical structures [Chen et al., 2018, Nielsen et al., 2021]. In light of these advancements, our proposed transposition-based approach for computing correlation over paired data presents a novel approach to computing ICC, potentially offering a faster and more efficient alternative. The full potential and utility of the transposition-based method for ICC computation, however, remain to be explored in future research.

R1-2 *Some important formulas should be numbered, such as in Definition and Theorem.*

Thank you for suggestion. We now numbered 23 major formulas.

Reviewer 2

R2-1 *Overall premise of the methodology is justified by the use of the Wasserstein distance to measure the distance between graphs. On page 11, the definition of the distance combines the 0D and 1D topological features via the sum of the squared distances. Can you please justify this definition?*

Thank you for suggestion. We added further explanations as follows.

In this study, to account for both 0D and 1D topological differences in net-

works, we use the sum of 0D and 1D Wasserstein distances between networks $\mathcal{X}_1$ and $\mathcal{X}_2$ as the topological distance between graphs

$$\mathcal{D}(\mathcal{X}_1, \mathcal{X}_2) = D_{W0}^2(P_1, P_2) + D_{W1}^2(P_1, P_2). \tag{1}$$

The equal weights of the form (1) were used for the following reasons. Through the birth-death decomposition, a weighted graph can be topologically characterized by 0D and 1D features, with no higher-dimensional features present. However, it is unclear which feature contributes the most. Equal weighting of 0D and 1D features ensures a balanced representation without bias towards either type of feature.

Let $\varnothing$ denote a graph with zero edge weights. Then, due to the birth-death decomposition, we have

$$\mathcal{D}(\mathcal{X}_1, \varnothing) = \sum_{i<j} (w_{ij}^1)^2, \quad \mathcal{D}(\varnothing, \mathcal{X}_2) = \sum_{i<j} (w_{ij}^2)^2,$$

where the squared sums of all the edge weights make the interpretation straightforward. If unequal weighting is used, these relationships do not hold. Further, the Wasserstein distances D_{W0} and D_{W1} are equivalent to the Euclidean distances in a convex set (the justification is given in the proof to Theorem 4 given in Appendix). Therefore, squared distance is a more natural choice that satisfies the triangle inequality

$$\mathcal{D}(\mathcal{X}_1, \mathcal{X}_3) \leq \mathcal{D}(\mathcal{X}_1, \mathcal{X}_2) + \mathcal{D}(\mathcal{X}_2, \mathcal{X}_3),$$

thus qualifying as a metric.

R2-1 *Sections 5.1 and 5.2 summarize the simulation results for "Testing for false positives" (5.1) and "Testing for false negatives" (5.2). Is my understanding incorrect that the proposed method shows worse performance than the k-means and hierarchical clustering?*

We apologize for any confusion. When there are no topological differences between groups, a higher clustering accuracy should be interpreted as a sign of false positives. Consequently, a clustering method that exhibits higher accuracy in such scenarios is inaccurately clustering the groups, erroneously identifying distinctions where none should exist. Therefore, the ostensibly lower clustering accuracy of the Wasserstein distance method actually reflects a more accurate performance in such scenarios. The sections have been revised to improve clarity as follows.

Testing for false positives

In the experiment depicted in Figure 6, we evaluated the occurrence of false positives in scenarios devoid of topological differences. All groups, derived from Group 1 through rotations, are topologically identical. Hence, any detected

differences are false positives. While k-means clustering exhibited an accuracy of 0.90 ± 0.15, and hierarchical clustering achieved perfect accuracy (1.00), these methods reported significant false positives, erroneously categorizing the groups as distinct clusters. The absence of inherent topological differences between the groups implies that higher clustering accuracy is indicative of false positive results. Conversely, topological clustering, with a lower accuracy of 0.53 ± 0.08, demonstrated a reduced tendency for false positive reporting in the absence of topological differences.

Testing for false negatives

Figure 7 presents our test for false negatives, featuring groups with varying numbers of cycles and distinct topologies. In this scenario, topological differences should be detectable. Here, k-means clustering recorded an accuracy of 0.83 ± 0.16, and hierarchical clustering again reported perfect accuracy. Notably, topological clustering attained a high accuracy of 0.98 ± 0.09. Separating topological from geometric signals is challenging; the presence of topological differences often coincides with geometric variations, which can influence the performance of all tested methods.

In summary, while traditional clustering methods based on geometric distances are prone to a significant number of false positives, making them less suitable for topological learning tasks, the proposed Wasserstein distance-based approach demonstrates superior performance. This method excels in minimizing both false positives and false negatives, as evidenced by our tests. Its effectiveness is particularly noteworthy in topological learning tasks, where discerning topological rather than geometric distinctions is crucial.

R2-1 *Sections 6.2 and 6.3 seem to be misplaced as they describe the methods used and not the data application.*

We moved the sections to the method section. They are now in section 5.

R2-1 *Overall, the manuscript is written in a very technical way introducing several advanced mathematical concepts. An algorithmic summary of the steps necessary to apply the proposed method to the rs-fMRI data would add to a greater accessibility for a wider audience.*

Thank you for suggestion. An algorithm summary is presented in a schematic flow chart (Figure 1), accompanied by detailed, step-by-step explanations below. Each section corresponds to one step within the algorithm.

The proposed method for estimating topological state space is based on the topological clustering of a collection of graphs (Figure 1). The initial step involves a birth-death decomposition of a weighted graph, leading to the generation of sorted birth and death sets (Section 3.3). The second step entails calculating the topological distance: between birth sets to obtain the 0D topo-

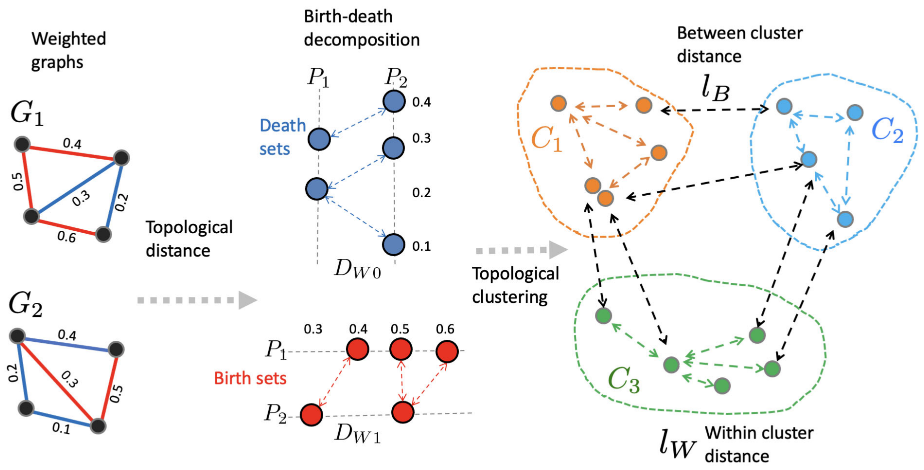

Figure 1: Proposed topological clustering pipeline used in estimating the state space. Given two weighted graphs G_1, G_2, we first perform the birth-death decomposition and partition the edges into sorted birth and death sets (section 3.3). The 0D topological distance D_{W0} between birth values quantifies discrepancies in connected components (section 4.1). The 1D topological distance D_{W1} between death values quantifies discrepancies in cycles (section 4.1). The combined distance $\mathcal{D} = D_{W0}^2 + D_{W1}^2$ is used in computing the within-cluster distance l_W between graphs. Topological clustering is performed by minimizing l_W over all possible cluster labels $C_1, \cdots, C_k$ (section 4.3).

logical distance d_0, and between death sets to ascertain the 1D topological distance d_1 (Section 4.1). The third step involves computing the within-cluster distance l_W among a collection of graphs (Section 4.3). Subsequently, we demonstrate the equivalence of topological clustering with k-means clustering in a high-dimensional convex set, employing k-means clustering routines for optimization.

R2-1 *Some of the more technical material reviewing the existing concepts and methods can be moved to an appendix.*

We removed all the proofs to the Theorems. We further deleted unnecessary mathematical details or simplified explanations.

References

M. Aktas, E. Akbas, and A. Fatmaoui. Persistence homology of networks: methods and applications. *Applied Network Science*, 4:1–28, 2019.

E. Allen, E. Damaraju, S. Plis, E. Erhardt, T. Eichele, and V. Calhoun. Tracking

whole-brain connectivity dynamics in the resting state. *Cerebral cortex*, 24: 663–676, 2014.

J. Billings, M. Saggar, J. Hlinka, S. Keilholz, and G. Petri. Simplicial and topological descriptions of human brain dynamics. *Network Neuroscience*, 5: 549–568, 2021.

B. Cai, P. Zille, J. Stephen, T. Wilson, V. Calhoun, and Y. Wang. Estimation of dynamic sparse connectivity patterns from resting state fMRI. *IEEE Transactions on Medical Imaging*, 37:1224–1234, 2018.

V. Calhoun and T. Adali. Time-varying brain connectivity in fMRI data: whole-brain data-driven approaches for capturing and characterizing dynamic states. *IEEE Signal Processing Magazine*, 33:52–66, 2016.

L. Caputi, A. Pidnebesna, and J. Hlinka. Promises and pitfalls of topological data analysis for brain connectivity analysis. *NeuroImage*, 238:118245, 2021.

C. Chen, X. Ni, Q. Bai, and Y. Wang. A topological regularizer for classifiers via persistent homology. In *The 22nd International Conference on Artificial Intelligence and Statistics*, pages 2573–2582. PMLR, 2019.

G. Chen, P. Taylor, S. Haller, K. Kircanski, J. Stoddard, D. Pine, E. Leibenluft, M. Brotman, and R. Cox. Intraclass correlation: Improved modeling approaches and applications for neuroimaging. *Human brain mapping*, 39: 1187–1206, 2018.

M. Chung, C. Ramos, F. De Paiva, J. Mathis, V. Prabhakaran, V. Nair, M. Meyerand, B. Hermann, J. Binder, and A. Struck. Unified topological inference for brain networks in temporal lobe epilepsy using the Wasserstein distance. *NeuroImage*, 284:120436, 2023.

J. Cole, R. Farmer, E. Rees, H. Johnson, C. Frost, R. Scahill, and N. Hobbs. Test-retest reliability of diffusion tensor imaging in HuntingtonÕs disease. *PLoS Currents*, 6, 2014.

M. Cousineau, P.-M. Jodoin, E. Garyfallidis, M.-A. Côté, F. Morency, V. Rozanski, M. GrandÕMaison, B. Bedell, and M. Descoteaux. A test-retest study on Parkinson's PPMI dataset yields statistically significant white matter fascicles. *NeuroImage: Clinical*, 16:222–233, 2017.

D. Glahn, A. Winkler, P. Kochunov, L. Almasy, R. Duggirala, M. Carless, J. Curran, R. Olvera, A. Laird, and S. Smith. Genetic control over the resting brain. *Proceedings of the National Academy of Sciences*, 107:1223–1228, 2010.

S. Gupta, X. Hu, J. Kaan, M. Jin, M. Mpoy, K. Chung, G. Singh, M. Saltz, T. Kurc, J. Saltz, et al. Learning topological interactions for multi-class medical image segmentation. In *European Conference on Computer Vision*, pages 701–718, 2022.

A. Haimovici, E. Tagliazucchi, P. Balenzuela, and H. Laufs. On wakefulness fluctuations as a source of BOLD functional connectivity dynamics. *Scientific Reports*, 7:5908, 2017.

C. Hofer, R. Kwitt, M. Niethammer, and M. Dixit. Connectivity-optimized representation learning via persistent homology. In *International Conference on Machine Learning*, pages 2751–2760, 2019.

X. Hu, F. Li, D. Samaras, and C. Chen. Topology-preserving deep image segmentation. In *Advances in Neural Information Processing Systems*, pages 5657–5668, 2019.

S.-G. Huang, S.-T. Samdin, C. Ting, H. Ombao, and M. Chung. Statistical model for dynamically-changing correlation matrices with application to brain connectivity. *Journal of Neuroscience Methods*, 331:108480, 2020.

A. Khalid, B. Kim, M. Chung, J. Ye, and D. Jeon. Tracing the evolution of multi-scale functional networks in a mouse model of depression using persistent brain network homology. *NeuroImage*, 101:351–363, 2014.

M. Korgaonkar, K. Ram, L. Williams, J. Gatt, and S. Grieve. Establishing the resting state default mode network derived from functional magnetic resonance imaging tasks as an endophenotype: a twins study. *Human brain mapping*, 35:3893–3902, 2014.

X.-H. Liao, M.-R. Xia, T. Xu, Z.-J. Dai, X.-Y. Cao, H.-J. Niu, X.-N. Zuo, Y.-F. Zang, and Y. He. Functional brain hubs and their test–retest reliability: a multiband resting-state functional mri study. *NeuroImage*, 83:969–982, 2013.

M. Lin, K. Zepf, A. Christensen, Z. Bashir, M. Svendsen, M. Tolsgaard, and A. Feragen. DTU-Net: Learning topological similarity for curvilinear structure segmentation. In *International Conference on Information Processing in Medical Imaging*, pages 654–666, 2023.

D. Lykken, A. Tellegen, and W. Iacono. EEG spectra in twins: Evidence for a neglected mechanism of genetic determination. *Physiological Psychology*, 10: 60–65, 1982.

N. Nielsen, W. Smink, and J.-P. Fox. Small and negative correlations among clustered observations: Limitations of the linear mixed effects model. *Behaviormetrika*, 48:51–77, 2021.

G. Petri, P. Expert, F. Turkheimer, R. Carhart-Harris, D. Nutt, P. Hellyer, and F. Vaccarino. Homological scaffolds of brain functional networks. *Journal of The Royal Society Interface*, 11:20140873, 2014.

E. Pfaehler, L. Mesotten, G. Kramer, M. Thomeer, K. Vanhove, J. de Jong, P. Adriaensens, O. S. Hoekstra, and R. Boellaard. Repeatability of two semi-automatic artificial intelligence approaches for tumor segmentation in PET. *EJNMMI research*, 11:1–11, 2021.

B. Rosenberg, E. Mennigen, M. Monti, and R. Kaiser. Functional segregation of human brain networks across the lifespan: an exploratory analysis of static and dynamic resting-state functional connectivity. *Frontiers in Neuroscience*, 14:561594, 2020.

P. Sarker, N. Zaman, J. Ong, P. Paladugu, M. Aldred, E. Waisberg, A. Lee, and A. Tavakkoli. Test–retest reliability of virtual reality devices in quantifying for relative afferent pupillary defect. *Translational Vision Science & Technology*, 12:2, 2023.

S. Shakil, C.-H. Lee, and S. Keilholz. Evaluation of sliding window correlation performance for characterizing dynamic functional connectivity and brain states. *NeuroImage*, 133:111–128, 2016.

A. Sizemore, C. Giusti, A. Kahn, J. Vettel, R. Betzel, and D. Bassett. Cliques and cavities in the human connectome. *Journal of computational neuroscience*, 44:115–145, 2018.

A. Sizemore, J. Phillips-Cremins, R. Ghrist, and D. Bassett. The importance of the whole: topological data analysis for the network neuroscientist. *Network Neuroscience*, 3:656–673, 2019.

J. Solís-Lemus, T. Baptiste, R. Barrows, C. Sillett, A. Gharaviri, G. Raffaele, O. Razeghi, M. Strocchi, I. Sim, and I. Kotadia. Evaluation of an open-source pipeline to create patient-specific left atrial models: A reproducibility study. *Computers in Biology and Medicine*, 162:107009, 2023.

F. Vaccarino, U. Fugacci, and S. Scaramuccia. Persistent homology: A topological tool for higher-interaction systems. In *Higher-Order Systems*, pages 97–139. 2022.

B. Wan, Ş. Bayrak, T. Xu, H. Schaare, R. Bethlehem, B. Bernhardt, and S. Valk. Heritability and cross-species comparisons of human cortical functional organization asymmetry. *Elife*, 11:e77215, 2022.

J. Xing, J. Jia, X. Wu, and L. Kuang. A spatiotemporal brain network analysis of alzheimer's disease based on persistent homology. *Frontiers in aging neuroscience*, 14:788571, 2022.

J. Xu, X. Yin, H. Ge, Y. Han, Z. Pang, B. Liu, S. Liu, and K. Friston. Heritability of the effective connectivity in the resting-state default mode network. *Cerebral Cortex*, 27:5626–5634, 2017.

Z. Zhang, M. Descoteaux, J. Zhang, G. Girard, M. Chamberland, D. Dunson, A. Srivastava, and H. Zhu. Mapping population-based structural connectomes. *NeuroImage*, 172:130–145, 2018.
